# Genetic Susceptibility to Acute Viral Bronchiolitis

**DOI:** 10.1093/infdis/jiae467

**Published:** 2024-09-20

**Authors:** Anu Pasanen, Minna K Karjalainen, Matti Korppi, Mikko Hallman, Mika Rämet, for the Research Project FinnGen, Aarno Palotie, Mark Daly, Bridget Riley-Gills, Howard Jacob, Coralie Viollet, Slavé Petrovski, Chia-Yen Chen, Sally John, George Okafo, Robert Plenge, Joseph Maranville, Mark McCarthy, Rion Pendergrass, Margaret G Ehm, Kirsi Auro, Simonne Longerich, Anders Mälarstig, Anna Vlahiotis, Katherine Klinger, Clement Chatelain, Matthias Gossel, Karol Estrada, Robert Graham, Dawn Waterworth, Chris O'Donnell, Nicole Renaud, Tomi P Mäkelä, Jaakko Kaprio, Minna Ruddock, Petri Virolainen, Antti Hakanen, Terhi Kilpi, Markus Perola, Jukka Partanen, Taneli Raivio, Jani Tikkanen, Raisa Serpi, Kati Kristiansson, Veli-Matti Kosma, Jari Laukkanen, Marco Hautalahti, Outi Tuovila, Jeffrey Waring, Bridget Riley-Gillis, Fedik Rahimov, Ioanna Tachmazidou, Chia-Yen Chen, Zhihao Ding, Marc Jung, Hanati Tuoken, Shameek Biswas, Rion Pendergrass, Margaret G Ehm, David Pulford, Neha Raghavan, Adriana Huertas-Vazquez, Jae-Hoon Sul, Anders Mälarstig, Xinli Hu, Åsa Hedman, Katherine Klinger, Robert Graham, Dawn Waterworth, Nicole Renaud, Ma'en Obeidat, Jonathan Chung, Jonas Zierer, Mari Niemi, Samuli Ripatti, Johanna Schleutker, Markus Perola, Mikko Arvas, Olli Carpén, Reetta Hinttala, Johannes Kettunen, Arto Mannermaa, Katriina Aalto-Setälä, Mika Kähönen, Jari Laukkanen, Johanna Mäkelä, Reetta Kälviäinen, Valtteri Julkunen, Hilkka Soininen, Anne Remes, Mikko Hiltunen, Jukka Peltola, Minna Raivio, Pentti Tienari, Juha Rinne, Roosa Kallionpää, Juulia Partanen, Adam Ziemann, Nizar Smaoui, Anne Lehtonen, Susan Eaton, Heiko Runz, Sanni Lahdenperä, Shameek Biswas, Natalie Bowers, Edmond Teng, Rion Pendergrass, Fanli Xu, David Pulford, Kirsi Auro, Laura Addis, John Eicher, Qingqin S Li, Karen He, Ekaterina Khramtsova, Neha Raghavan, Martti Färkkilä, Jukka Koskela, Sampsa Pikkarainen, Airi Jussila, Katri Kaukinen, Timo Blomster, Mikko Kiviniemi, Markku Voutilainen, Mark Daly, Jeffrey Waring, Nizar Smaoui, Fedik Rahimov, Anne Lehtonen, Tim Lu, Natalie Bowers, Rion Pendergrass, Linda McCarthy, Amy Hart, Meijian Guan, Jason Miller, Kirsi Kalpala, Melissa Miller, Xinli Hu, Kari Eklund, Antti Palomäki, Pia Isomäki, Laura Pirilä, Oili Kaipiainen-Seppänen, Johanna Huhtakangas, Nina Mars, Jeffrey Waring, Fedik Rahimov, Apinya Lertratanakul, Nizar Smaoui, Anne Lehtonen, Coralie Viollet, Marla Hochfeld, Natalie Bowers, Rion Pendergrass, Jorge Esparza Gordillo, Kirsi Auro, Dawn Waterworth, Fabiana Farias, Kirsi Kalpala, Nan Bing, Xinli Hu, Tarja Laitinen, Margit Pelkonen, Paula Kauppi, Hannu Kankaanranta, Terttu Harju, Riitta Lahesmaa, Nizar Smaoui, Coralie Viollet, Susan Eaton, Hubert Chen, Rion Pendergrass, Natalie Bowers, Joanna Betts, Kirsi Auro, Rajashree Mishra, Majd Mouded, Debby Ngo, Teemu Niiranen, Felix Vaura, Veikko Salomaa, Kaj Metsärinne, Jenni Aittokallio, Mika Kähönen, Jussi Hernesniemi, Daniel Gordin, Juha Sinisalo, Marja-Riitta Taskinen, Tiinamaija Tuomi, Timo Hiltunen, Jari Laukkanen, Amanda Elliott, Mary Pat Reeve, Sanni Ruotsalainen, Dirk Paul, Natalie Bowers, Rion Pendergrass, Audrey Chu, Kirsi Auro, Dermot Reilly, Mike Mendelson, Jaakko Parkkinen, Melissa Miller, Tuomo Meretoja, Heikki Joensuu, Olli Carpén, Johanna Mattson, Eveliina Salminen, Annika Auranen, Peeter Karihtala, Päivi Auvinen, Klaus Elenius, Johanna Schleutker, Esa Pitkänen, Nina Mars, Mark Daly, Relja Popovic, Jeffrey Waring, Bridget Riley-Gillis, Anne Lehtonen, Margarete Fabre, Jennifer Schutzman, Natalie Bowers, Rion Pendergrass, Diptee Kulkarni, Kirsi Auro, Alessandro Porello, Andrey Loboda, Heli Lehtonen, Stefan McDonough, Sauli Vuoti, Kai Kaarniranta, Joni A Turunen, Terhi Ollila, Hannu Uusitalo, Juha Karjalainen, Esa Pitkänen, Mengzhen Liu, Heiko Runz, Stephanie Loomis, Erich Strauss, Natalie Bowers, Hao Chen, Rion Pendergrass, Kaisa Tasanen, Laura Huilaja, Katariina Hannula-Jouppi, Teea Salmi, Sirkku Peltonen, Leena Koulu, Nizar Smaoui, Fedik Rahimov, Anne Lehtonen, David Choy, Rion Pendergrass, Dawn Waterworth, Kirsi Kalpala, Ying Wu, Pirkko Pussinen, Aino Salminen, Tuula Salo, David Rice, Pekka Nieminen, Ulla Palotie, Maria Siponen, Liisa Suominen, Päivi Mäntylä, Ulvi Gursoy, Vuokko Anttonen, Kirsi Sipilä, Rion Pendergrass, Hannele Laivuori, Venla Kurra, Laura Kotaniemi-Talonen, Oskari Heikinheimo, Ilkka Kalliala, Lauri Aaltonen, Varpu Jokimaa, Johannes Kettunen, Marja Vääräsmäki, Outi Uimari, Laure Morin-Papunen, Maarit Niinimäki, Terhi Piltonen, Katja Kivinen, Elisabeth Widen, Taru Tukiainen, Mary Pat Reeve, Mark Daly, Niko Välimäki, Eija Laakkonen, Jaakko Tyrmi, Heidi Silven, Eeva Sliz, Riikka Arffman, Susanna Savukoski, Triin Laisk, Natalia Pujol, Mengzhen Liu, Bridget Riley-Gillis, Rion Pendergrass, Janet Kumar, Kirsi Auro, Iiris Hovatta, Chia-Yen Chen, Erkki Isometsä, Hanna Ollila, Jaana Suvisaari, Antti Mäkitie, Argyro Bizaki-Vallaskangas, Sanna Toppila-Salmi, Tytti Willberg, Elmo Saarentaus, Antti Aarnisalo, Eveliina Salminen, Elisa Rahikkala, Johannes Kettunen, Kristiina Aittomäki, Fredrik Åberg, Mitja Kurki, Samuli Ripatti, Mark Daly, Juha Karjalainen, Aki Havulinna, Juha Mehtonen, Priit Palta, Shabbeer Hassan, Pietro Della Briotta Parolo, Wei Zhou, Mutaamba Maasha, Shabbeer Hassan, Susanna Lemmelä, Manuel Rivas, Aarno Palotie, Aoxing Liu, Arto Lehisto, Andrea Ganna, Vincent Llorens, Hannele Laivuori, Taru Tukiainen, Mary Pat Reeve, Henrike Heyne, Nina Mars, Joel Rämö, Elmo Saarentaus, Hanna Ollila, Rodos Rodosthenous, Satu Strausz, Tuula Palotie, Kimmo Palin, Javier Garcia-Tabuenca, Harri Siirtola, Tuomo Kiiskinen, Jiwoo Lee, Kristin Tsuo, Amanda Elliott, Kati Kristiansson, Mikko Arvas, Kati Hyvärinen, Jarmo Ritari, Olli Carpén, Johannes Kettunen, Katri Pylkäs, Eeva Sliz, Minna Karjalainen, Tuomo Mantere, Eeva Kangasniemi, Sami Heikkinen, Arto Mannermaa, Eija Laakkonen, Nina Pitkänen, Samuel Lessard, Clément Chatelain, Lila Kallio, Tiina Wahlfors, Jukka Partanen, Eero Punkka, Raisa Serpi, Sanna Siltanen, Veli-Matti Kosma, Teijo Kuopio, Anu Jalanko, Huei-Yi Shen, Risto Kajanne, Mervi Aavikko, Helen Cooper, Denise öller, Rasko Leinonen, Henna Palin, Malla-Maria Linna, Mitja Kurki, Juha Karjalainen, Pietro Della Briotta Parolo, Arto Lehisto, Juha Mehtonen, Wei Zhou, Masahiro Kanai, Mutaamba Maasha, Zhili Zheng, Hannele Laivuori, Aki Havulinna, Susanna Lemmelä, Tuomo Kiiskinen, L Elisa Lahtela, Mari Kaunisto, Elina Kilpeläinen, Timo P Sipilä, Oluwaseun Alexander Dada, Awaisa Ghazal, Anastasia Kytölä, Rigbe Weldatsadik, Sanni Ruotsalainen, Kati Donner, Timo P Sipilä, Anu Loukola, Päivi Laiho, Tuuli Sistonen, Essi Kaiharju, Markku Laukkanen, Elina Järvensivu, Sini Lähteenmäki, Lotta Männikkö, Regis Wong, Auli Toivola, Minna Brunfeldt, Hannele Mattsson, Kati Kristiansson, Susanna Lemmelä, Sami Koskelainen, Tero Hiekkalinna, Teemu Paajanen, Priit Palta, Shuang Luo, Tarja Laitinen, Mary Pat Reeve, Shanmukha Sampath Padmanabhuni, Marianna Niemi, Harri Siirtola, Javier Gracia-Tabuenca, Mika Helminen, Tiina Luukkaala, Iida Vähätalo, Jyrki Tammerluoto, Marco Hautalahti, Johanna Mäkelä, Sarah Smith, Tom Southerington, Petri Lehto

**Affiliations:** Research Unit of Clinical Medicine, University of Oulu; Medical Research Center Oulu, Oulu University Hospital and University of Oulu; Department of Children and Adolescents, Oulu University Hospital; Medical Research Center Oulu, Oulu University Hospital and University of Oulu; Research Unit of Population Health, Faculty of Medicine, University of Oulu; Child Health Research Center, Tampere University and University Hospital; Faculty of Medicine and Health Technology, Tampere University, Finland; Research Unit of Clinical Medicine, University of Oulu; Medical Research Center Oulu, Oulu University Hospital and University of Oulu; Department of Children and Adolescents, Oulu University Hospital; Research Unit of Clinical Medicine, University of Oulu; Medical Research Center Oulu, Oulu University Hospital and University of Oulu; Department of Children and Adolescents, Oulu University Hospital; Faculty of Medicine and Health Technology, Tampere University, Finland

**Keywords:** asthma, bronchiolitis, *CDHR3*, genetic risk factors, *GSDMB*

## Abstract

**Background:**

Acute viral bronchiolitis is a major cause of infant hospitalizations worldwide. Childhood bronchiolitis is considered a risk factor for asthma, suggesting shared genetic factors and biological pathways. Genetic risk loci may provide new insights into disease pathogenesis.

**Methods:**

We conducted a genome-wide association study to examine the genetic contributions to bronchiolitis susceptibility in the FinnGen project data. We analyzed 1465 infants hospitalized for bronchiolitis who were <2 years of age and 356 404 individuals without a history of acute lower respiratory infections.

**Results:**

The genome-wide association study identified associations (*P* < 5 × 10^−8^) for variants in gasdermin B (*GSDMB*) and a missense variant in cadherin-related family member 3 (*CDHR3*). Children with bronchiolitis in infancy were more likely to develop asthma later in life as compared with controls. The 2 associated loci were previously linked to asthma and susceptibility to wheezing illness by causative agents other than respiratory syncytial virus (RSV). The identified loci were associated with overall bronchiolitis, with larger effects in non-RSV than RSV-induced infection.

**Conclusions:**

Our results suggest that genetic variants in *CDHR3* and *GSDMB* modulate susceptibility to bronchiolitis, especially when caused by viruses other than RSV. Severe bronchiolitis in infancy may trigger the development of asthma in genetically susceptible individuals, or it could be a marker of genetic predisposition to asthma.

## Teaser

We identified bronchiolitis-associated loci in gasdermin B (*GSDMB*) and cadherin-related family member 3 (*CDHR3*) by genome-wide association study. Our analysis discovered associations with overall bronchiolitis, with larger effects in infection without respiratory syncytial virus than with it.

**(See the Editorial Commentary by Snyder and Hartert on pages e186–8.)**

Acute viral bronchiolitis is a common lower respiratory infection (LRI) that affects infants and young children worldwide. Bronchiolitis is most often caused by respiratory syncytial virus (RSV), followed by other causative viruses, such as rhinovirus and metapneumovirus [1–3]. RSV is the major causal agent at <6 months of age and rhinovirus at >12 months [4]. Most infants with viral respiratory infections have a mild upper airway disease with cold-like symptoms, including low-grade fever, runny nose, and nasal congestion. Some infants present with severe symptoms (eg, respiratory distress, fatigue) that require medical attention and may develop into a condition that is life-threatening, necessitating intensive care. Bronchiolitis is the leading cause of LRI-related hospitalizations of children younger than 2 years in developed countries [3, 5, 6].

There is no curative treatment for bronchiolitis, and the symptomatic treatment focuses on supporting oxygen supply and maintaining hydration [7]. A maternal RSV vaccine may be given during the third trimester of pregnancy in the RSV season to protect the newborn from severe RSV illness [8]. For children at high risk of developing severe RSV bronchiolitis, immunoprophylaxis with the monoclonal antibody palivizumab or nirsevimab can be used to prevent severe illness [1, 9]. In many countries, nirsevimab or maternal RSV vaccine is recommended for the prevention of RSV lower respiratory disease in all infants during their first RSV season [9, 10]. Moreover, some infants with rhinovirus bronchiolitis may benefit from systemic corticosteroids [2, 7].

The most important risk factors for severe bronchiolitis are premature birth, young age (younger than 2–3 months), chronic lung disease of premature infants, immunodeficiency, and congenital heart disease [3]. However, the known risk factors do not sufficiently explain bronchiolitis severity, as most infants hospitalized with bronchiolitis have no identified predisposing conditions. This suggests that genetic and other currently unidentified factors play a role in bronchiolitis severity.

The contribution of genetic variation to bronchiolitis severity is approximately 22% to 40% based on heritability estimates obtained from European twin studies [11, 12]. Previous studies of bronchiolitis genetics include several investigations of candidate gene polymorphisms with plausible roles in bronchiolitis pathophysiology [13]. The innate immune response has been shown to affect bronchiolitis severity, and variants in immune system genes have been of particular interest in candidate gene studies of bronchiolitis [14–16]. Variants suggested to be associated with bronchiolitis or RSV bronchiolitis include polymorphisms in genes encoding surfactant proteins (*SFTPA1*, *SFTPA2*, *SFTPD*) and genes *NKG2D* and *TLR4* [17–20]. Variants suggested to be associated with rhinovirus-induced wheezing or non-RSV bronchiolitis include variants in a genomic region near 17q21 and cadherin-related family member 3 (*CDHR3*) [21–23]. There are a few hypothesis-free assessments of bronchiolitis genetics, and these genome-wide association studies (GWASs) have found suggestive associations with bronchiolitis [24–26]. A whole exome sequencing study suggested associations near genes such as *OR13C5*, *HLA-DQA1*, and *MUC4* with RSV bronchiolitis [27]. Altogether, findings from most genetic studies of bronchiolitis were based on relatively small sample sizes, and their results have rarely been replicated in subsequent studies. Thus, the genetic background of bronchiolitis remains largely undercharacterized. A better characterization of host genetic factors could provide insight into the disease process and help recognize high-risk infants.

There is a link between severe viral bronchiolitis in infancy and later recurrent wheezing and asthma [1, 2]. While occasional wheezing symptoms in childhood are common, they may also represent the first manifestation of asthma [28]. Severe viral bronchiolitis could activate pathways that lead to the development of asthma [13, 29]. There are several inflammatory pathways associated with asthma, and multiple asthma-associated genetic loci are known [30]. Comparison with bronchiolitis has been limited by incomplete knowledge of the underlying genetic associations with bronchiolitis. Moreover, recent research suggests more complex interactions among the host, causative respiratory viruses, and subsequent recurrent wheeze or asthmatic symptoms [29, 31]. Genetic variants in genes (eg, *TLR4*, *VDR*, and *CCR5*) have been suggested to be associated with RSV bronchiolitis and the risk of asthma [13, 32]. Additionally, genetic variants in genomic region 17q21 and *CDHR3* showed suggestive associations with non-RSV LRI phenotypes and later asthma [21, 22, 33].

The aim of the present study was to identify genetic variants that may predispose infants <2 years of age to severe bronchiolitis by utilizing large-scale genetic data. Moreover, we aimed to assess genetic associations with bronchiolitis linked to RSV and non-RSV infection and determine the effect of these variants on the risk of developing asthma.

## METHODS

### Ethical Considerations

Patients and controls in FinnGen provided informed consent for biobank research per the Finnish Biobank Act. Alternatively, separate research cohorts—assembled before the Finnish Biobank Act came into effect (September 2013)—were based on study-specific consents and later transferred to the Finnish biobanks after approval by Fimea (Finnish Medicines Agency), the National Supervisory Authority for Welfare and Health. The entire FinnGen ethics statement with permit numbers is available in the supplementary appendix.

### Study Data

Data were used from FinnGen Preparatory Phase Data Freeze 9. The FinnGen research project (www.finngen.fi) coalesces genome information with digital health care data with the help of national personal identification numbers. The GWASs of bronchiolitis and its subgroups were conducted with data comprising 1456 cases and 356 404 controls.

Phenotypes were defined by the *International Classification of Diseases* entry in the Care Register for Health Care inpatient visits maintained by the Finnish Institute for Health and Welfare. For cases, we included individuals with *ICD-10* code J21 for acute bronchiolitis (J21.0, acute bronchiolitis due to RSV; J21.8, acute bronchiolitis due to other specified organisms; J21.90, acute obstructive bronchitis of infants; J21.99, unspecified acute bronchiolitis), *ICD-9* codes 4661A and 46 602 for unspecified acute bronchiolitis and bronchitis, and *ICD-8* entry 466.99 for acute bronchitis or bronchiolitis. For consistency with bronchiolitis phenotypes in previous studies, the upper age limit was set to 2 years. All cases were hospitalized with bronchiolitis. We further studied bronchiolitis subgroups stratified by RSV status and age. RSV and non-RSV bronchiolitis groups were formed with the *ICD-10* codes for which the RSV infections could be identified: we included individuals with *ICD-10* code J21.0 in the RSV bronchiolitis group and individuals with *ICD-10* code J21.8, J21.90, or J21.99 in the non-RSV bronchiolitis group. The age groups were <6 months, 0 to 1 years, 1 to 2 years, and 0 to 2 years. Virus-specific subgroups were analyzed with GWAS. Populations stratified by RSV status and age were used to follow up on asthma incidences in the specific subgroups and compare effect estimates among the groups. Controls were those without a history of acute LRIs (ie, no inpatient or outpatient visits due to LRIs).

### Genotyping, Imputation, and Quality Control

Genotyping of the FinnGen samples was done with Illumina and Affymetrix arrays (Illumina Inc and Thermo Fisher Scientific). Eligible samples had nonambiguous sex, Finnish ancestry, nonhigh missingness (<5%), and no excess heterozygosity (±4 SD). Exclusion criteria in variant quality control were high missingness (>2%), minor allele count <3, and deviation from Hardy-Weinberg equilibrium (*P* < 1e−6). Genotype imputation was done with Beagle 4.1, and a Finnish population-specific SISu reference panel was used. In postimputation quality control, variants with INFO <0.6 were excluded.

### Genome-wide Association Study

GWAS was conducted with the REGENIE whole genome regression model implemented in the FinnGen environment. GWAS covariates were age, sex, genotyping batch, and the first 10 principal components. We conducted a GWAS of bronchiolitis by any causal virus and 2 subgroup analyses stratified by the RSV status: RSV bronchiolitis GWAS and non-RSV bronchiolitis GWAS. In post-GWAS quality control, variants with a minor allele frequency of 1% were retained (n = 9 199 013).

### Prevalence of Asthma After Bronchiolitis

We used predefined phenotypes for asthma to investigate the incidence of asthma in bronchiolitis cases and controls within the FinnGen data and to confirm the well-established association between bronchiolitis and asthma. Asthma definition was based on the diagnosis codes of the Finnish version of the *International Statistical Classification of Diseases and Related Health Problems* (*ICD-10*, J45 and J46; *ICD-9*, 493; *ICD-8*, 493). We used Fisher exact test to assess the differences in asthma prevalence between RSV and non-RSV bronchiolitis and between bronchiolitis and controls.

### Expression Quantitative Trait Locus Analysis

We used colocalization analysis to assess if expression quantitative trait loci (eQTLs) and GWAS signals may share a genetic cause. We used cis-eQTL data from eQTL Catalogue release 6 [34] and Hyprcoloc [35] to test for colocalization in 500-kb windows around the index variants of the gasdermin B (*GSDMB*) and *CDHR3* loci. As suggested by prior knowledge of the bronchiolitis-infected cell types, the analysis was performed in blood, spleen, lung, monocytes, neutrophils, macrophages, B cells, natural killer cells, lymphoblastoid cells lines, CD4 and CD8 T cells, T regulatory cells, memory T regulatory cells, and follicular T helper (Th) cells, as well as Th1, Th2, and Th17 cells.

### Phenome-wide Association Study

In the phenome-wide association study (pheWAS), previous associations of the lead variants of the associated loci were screened in the 2408 phenotypes available in the FinnGen DF10 data via Finngen's DF10 browser (https://r10.finngen.fi/).

### Linkage Disequilibrium Score Regression Analysis

We used linkage disequilibrium score regression (LDSC) to estimate heritability and assess genetic correlations between bronchiolitis and other complex traits in a comprehensive set of 772 phenotypes based on GWASs on European populations, downloaded from the Integrative Epidemiology Unit’s OpenGWAS Project (https://gwas.mrcieu.ac.uk/) [36, 37]. European linkage disequilibrium scores available with the LDSC command line tool were utilized (https://github.com/bulik/ldsc) [36]. We used within-group corrected *P* value thresholds to assess the significance of correlation. Heritability was estimated on a liability scale with a sample and population prevalence of 0.5%.

## RESULTS

### Bronchiolitis Phenotypes and Asthma

Phenotypes of the 357 869 study individuals are summarized in Table 1. All cases were hospitalized for bronchiolitis, and most (59.9%) were <12 months of age. As expected, cases with RSV bronchiolitis were younger than infants with non-RSV bronchiolitis. Concordantly, the median age during the first bronchiolitis was lower in cases with RSV (0.34 years) as compared with cases with non-RSV (0.85 years) or overall bronchiolitis (0.79 years). Of note, most non-RSV bronchiolitis samples in the current data were cases with unspecified bronchiolitis and thus may include unrecorded cases of RSV. However, the proportions of RSV and non-RSV are in line with previously observed viral causes of bronchiolitis in Finland in cases younger than 6 months, 1 year, and 2 years of age (Table 1) [24, 38].

**Table 1.** Bronchiolitis Controls and Cases in the Overall Genome-wide Association Study (*ICD-8* to *ICD-10*) and Subanalysis Non-RSV and RSV Bronchiolitis (*ICD-10*)

	*ICD-8* to *ICD-10*		*ICD-10*	
	Controls	Bronchiolitis	Non-RSV Bronchiolitis	RSV Bronchiolitis
Controls	356 404	…	…	…
Cases	…	1465	484	263
Age, y				
Mean ± SD	*…*	0.85 ± 0.55	0.90 ± 0.54	0.48 ± 0.46
Median	…	0.79	0.85	0.34
Hospitalization for bronchiolitis	0	1465 (100)	484 (100)	263 (100)
Sex: male	155 622 (44)	717 (48.9)	233 (48.1)	116 (44)
Age of cases				
1–2 y	…	587 (40.1)	219 (45.2)	40 (15.2)
0–1 y	*…*	878 (59.9)	265 (54.8)	223 (84.4)
0–6 mo	*…*	447 (30.5)	129 (26.7)	171 (65.0)

Data are presented as No. (%) unless noted otherwise.

Abbreviations: *ICD*, *International Classification of Diseases*; RSV, respiratory syncytial virus.

We assessed whether severe bronchiolitis is associated with the risk of asthma in the current study population. As shown in Supplementary Table 1, the prevalence of asthma was substantially higher in individuals with bronchiolitis (38.6%) as compared with those with no history of LRIs (10.0%). The odds of asthma increased with age during bronchiolitis. We further detected higher odds of asthma development in individuals with non-RSV bronchiolitis (55,6%) as compared with individuals with RSV bronchiolitis (36.5%), and the differences in odds were similar across the tested age groups. The findings in the FinnGen data are consistent with previous reports of asthma after bronchiolitis in adolescence and adulthood [4, 39].

### Genetic Loci Associated With Bronchiolitis in the Genome-wide Investigation

**Figure 1.**
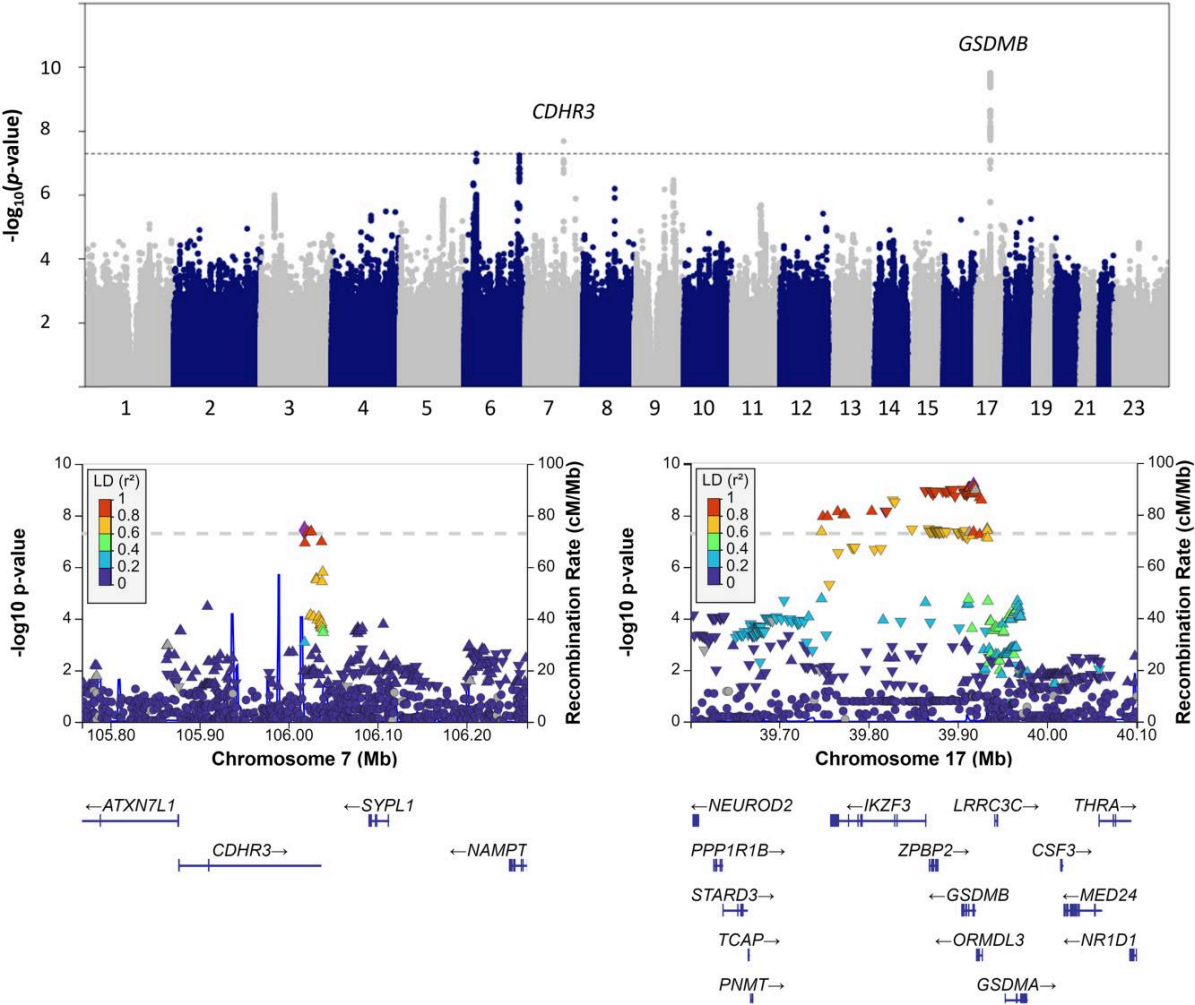
Manhattan plot and regional association plots of the genome-wide association study of bronchiolitis. The y- and x-axes show −log_10_  *P* values vs chromosomal positions of the tested variants. The dashed line denotes the threshold of genome-wide significance (*P* < 5 × 10^−8^).

Genome-wide association analysis was performed with 1465 infants admitted to the hospital with bronchiolitis (<2 years) and 356 404 controls without a history of LRIs. The GWASs of non-RSV and RSV bronchiolitis (subanalysis) were conducted with 484 and 263 cases, respectively, against the same set of controls. The lambda (0.5) values of 1.027, 0.94, and 0.801 in the GWASs of bronchiolitis, non-RSV bronchiolitis, and RSV bronchiolitis indicated no genome-wide inflation but suggested that the subanalysis could be underpowered. The GWAS of bronchiolitis by any causative virus found 2 associated loci (*P* < 5 × 10^−8^), located within *CDHR3* and *GSDMB* (Figure 1, Table 2). The age-stratified association statistics for these loci are shown in Supplementary Table 2. The effect sizes in the cases younger than 1 year and 6 months of age were in the same direction when compared with the main analysis.

**Table 2.** Loci Associated (*P* < 5e−8) With Bronchiolitis in the Genome-wide Association Study of 1465 Cases and 356 404 Controls

			Allele						
Chr	rsID	Position^a^	Effect	Other	*P* Value	Odds Ratio (95% CI)	Frequency	Locus	Most Severe Annotation
7	rs6967330	106018005	A	G	3.76e−08	1.256 (1.158–1.363)	0.268	*CDHR3*	Missense variant
17	rs9303281	39917793	A	G	7.45e−10	1.261 (1.171–1.358)	0.448	*GSDMB*	Noncoding transcript exon variant

Abbreviation: Chr, chromosome.

^a^Position as base pairs in Grch38 coordinates.

**Figure 2.**
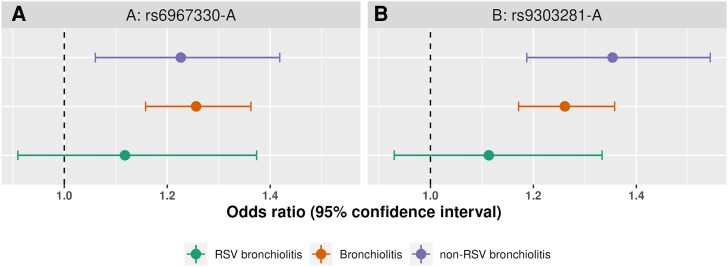
Effect sizes of the associated loci in the genome-wide association study of bronchiolitis and the subanalysis of RSV and non-RSV bronchiolitis. Effects are shown for the index variant in the (*A*) *CDHR3* and (*B*) *GSDMB* loci. RSV, respiratory syncytial virus.

*CDHR3* rs6967330 missense variant was previously associated with childhood-onset asthma [40], otitis media [41], and non-RSV bronchiolitis [22]. The transmembrane protein encoded by *CDHR3* is highly expressed in the airway epithelia and is a known receptor for rhinovirus C [42]. rs6967330-A increases the cell surface expression of CDHR3 in the airway epithelium, which is in turn associated with increased replication of rhinovirus C [43, 44]. In line with the known role of *CDHR3* in rhinovirus pathophysiology, our results found a nominal association in the analysis of non-RSV bronchiolitis (*P* = 6e−3) but not in the analysis of RSV bronchiolitis (*P* = .29). The effect size was smaller in RSV bronchiolitis when compared with overall and non-RSV bronchiolitis, although the differences were not statistically significant, likely due to a small sample size in the virus-specific analysis (Figure 2*A*).

There were several hundred associated variants in the *GSDMB* locus in high linkage disequilibrium spanning several genes, such as *IKZF3*, *ORMDL3*, and *LRRC3C. GSDMB* locus variants were previously associated with asthma, cardiovascular disease, and neutrophil counts in the GWAS Catalog [45]. Variants in the *GSDMB* locus were previously suggested to play a role in rhinovirus but not in RSV wheezing illness in children <3 years of age [21]. The current study showed an association with overall bronchiolitis (Table 2, Figure 1), and the virus-specific analysis found a suggestive association with non-RSV bronchiolitis (rs9303281, *P* = 6.2e−6) but no association with RSV bronchiolitis (rs9303281, *P* = .24). The effect size was largest in the non-RSV bronchiolitis (Figure 2*B*). Moreover, the non-RSV bronchiolitis effect was significantly larger than the effect in the RSV bronchiolitis GWAS.

### eQTL Analysis

We conducted colocalization analysis to assess if the bronchiolitis association in the *CDHR3* or *GSDMB* locus could be mediated by gene expression. A previous study detected that expression levels of *ORMDL3* and *GSDMB* were increased in rhinovirus-stimulated peripheral blood mononuclear cells, as compared with unstimulated ones [21]. The increase in expression due to exposure to rhinovirus was, however, not dependent on the genotype. We used eQTL data from other relevant tissues and more specific immune cell types to assess if the bronchiolitis association in the *GSDMB* locus could be modulated by genotype-specific gene expression. The analysis suggested that *GSDMB* locus variants were associated with the expression of *GSDMB* and *ORMDL* in cells or tissues, such as natural killer cells, B cells, spleen, and CD8 T cells (Supplementary Table 3). Increased gene expression was associated with an increased risk of bronchiolitis in all tested cell types and tissues. We did not detect colocalization for *CDHR3* in the tested tissue types.

### Subanalysis of RSV and Non-RSV Bronchiolitis

The subanalysis found suggestively associated loci (*P* < 1e−6) with non-RSV and RSV bronchiolitis (Supplementary Table 4). A variant in *SH3D19* showed borderline-significant association with overall bronchiolitis. *SH3D19* encodes SH3 domain containing 19, and the gene was previously associated with traits such as height and white blood cell counts in the GWAS Catalog [45]. Furthermore, the gene showed an association with bovine respiratory infections [46] and suggestive associations with asthma and hospitalization for COVID-19 [45].

### Phenome-wide Association Study

**Figure 3.**
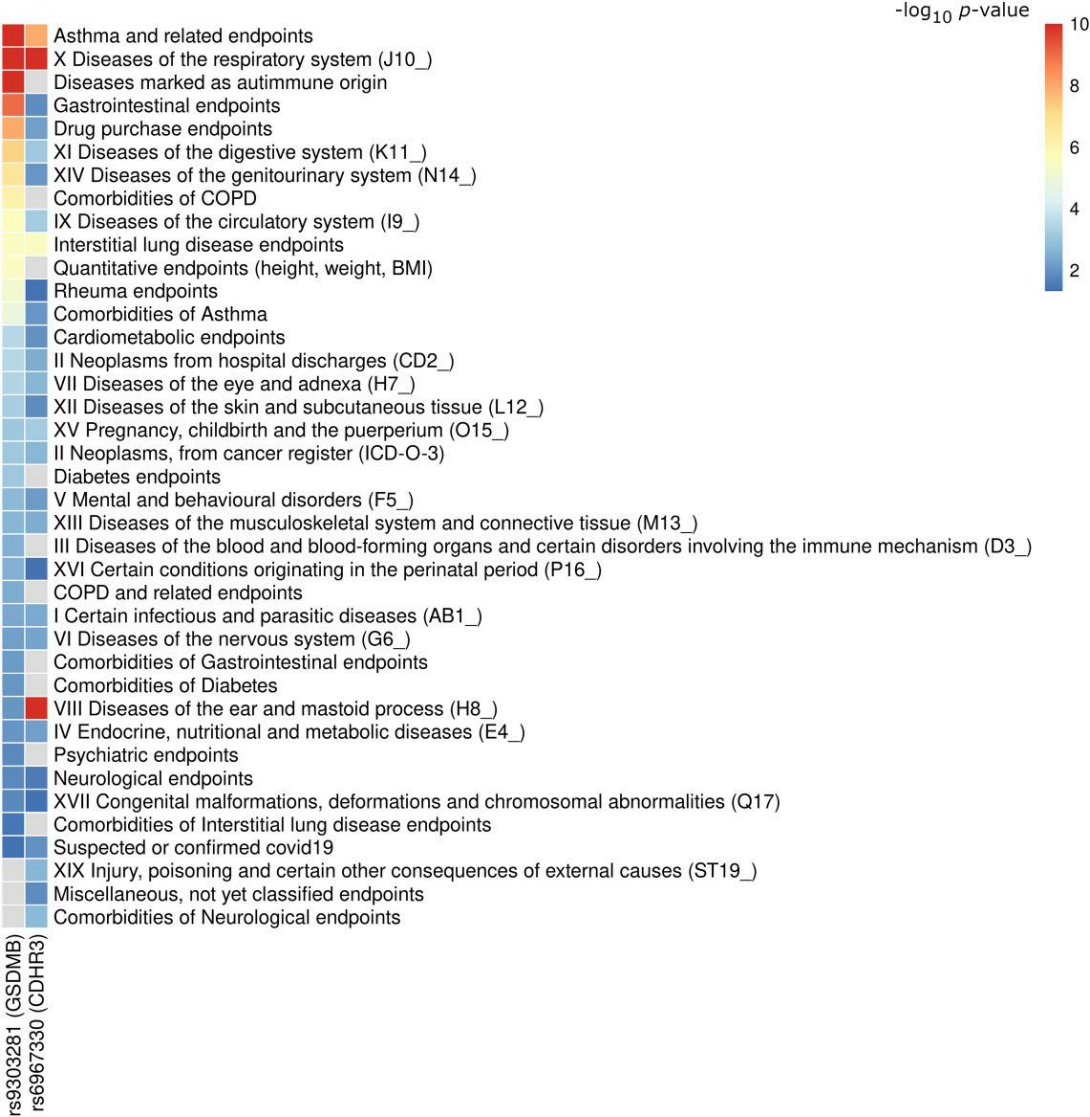
Associations of the bronchiolitis genome-wide association study lead single-nucleotide polymorphisms among the FinnGen DF10 phenotype categories. The association *P* value is based on the phenotype with the strongest within-category association. The associations are capped into a −log_10_  *P* value of 10.

We used pheWAS to evaluate the impact of bronchiolitis-associated genetic variants across other traits. The lead variants of the *CDHR3* and *GSDMB* loci showed significant associations with asthma and respiratory system–related phenotypes, assayed among the traits in the FinnGen DF10 (Figure 3, Supplementary Table 5).

### Genetic Correlation and Heritability

**Figure 4.**
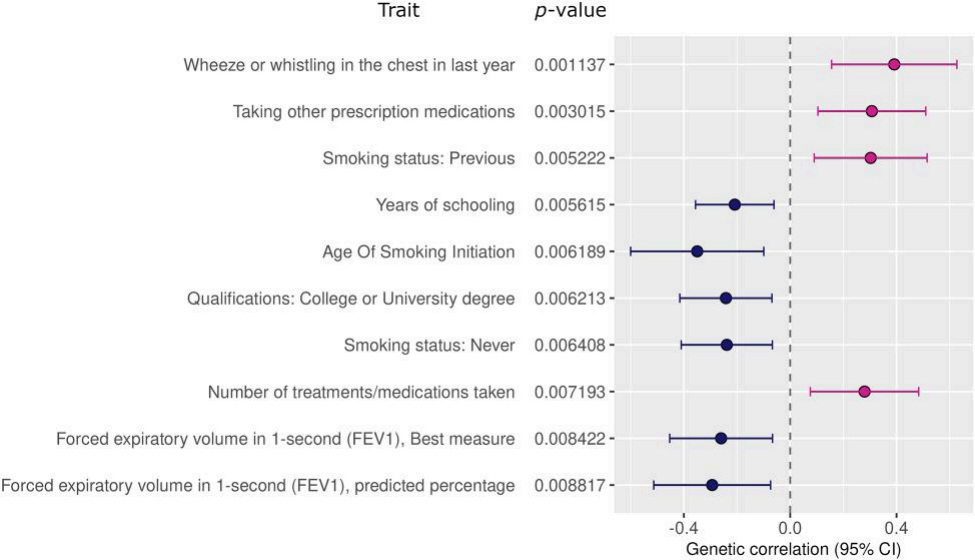
Genetic correlation between bronchiolitis and other complex traits. The figure shows the top 10 correlating traits. Bronchiolitis correlations were tested with data originating from 772 European genome-wide association studies obtained via the Integrative Epidemiology Unit’s OpenGWAS Project [37]. The reports describing the genome-wide association study data can be searched online in the OpenGWAS database (https://gwas.mrcieu.ac.uk/) with the identification numbers (GWAS_ID) provided in Supplementary Table 6.

We used LDSC to estimate heritability and shared genetic architecture of bronchiolitis and other complex traits with GWAS data from European populations [36, 37]. Wheezing showed a positive correlation with bronchiolitis, whereas smoking and spirometry measures of lung function appeared among the top traits with suggestive bronchiolitis correlations (Figure 4, Supplementary Table 6).

Bronchiolitis showed negative correlations with spirometry measures of normal respiratory function. It was previously shown that lung growth deficits shortly after birth are a risk factor for LRIs, especially due to RSV [47]. Our results suggest that bronchiolitis and decreased pulmonary function measured with spirometry may share genetic determinants. Childhood asthma was among the traits that showed genetic correlation with bronchiolitis (genetic correlation, *r*_g_ = 0.62), demonstrating shared genetic architecture between these complex phenotypes.

The LDSC single-nucleotide polymorphism–based heritability estimate of bronchiolitis on a total liability scale was 7.6%, with a sample and population prevalence of 0.5%. The estimate suggests that there are further genetic factors of susceptibility to bronchiolitis to be discovered. These could consist of additional common genetic variants that require a larger number of cases to be detected and rare genetic variants not captured by the genotyping chips in the current analysis.

## DISCUSSION

Epidemiologic studies have found increased rates of asthma among individuals with bronchiolitis in infancy [4, 39], and in the present study, conducted with the FinnGen data, we show similar associations between severe bronchiolitis and later asthma. Furthermore, the incidence of asthma was greater in individuals with non-RSV bronchiolitis as compared with RSV bronchiolitis. We detected 2 loci, *CDHR3* and *GSDMB*, that were associated with severe bronchiolitis in the GWAS. Supplementary analyses suggested that these loci mediate susceptibility to non-RSV rather than RSV bronchiolitis.

*CDHR3* and *GSDMB* loci were associated with asthma in previous studies and the pheWAS, and the *GSDMB* locus in the long arm of chromosome 17 (17q) is the strongest known asthma risk locus. We showed that regulatory variants in the *GSDMB* locus may influence *GSDMB* and *ORMDL3* expression in distinct immune cell types and that the genotype-dependent gene expression could mediate susceptibility to severe bronchiolitis. Increased gene expression in blood and inflammatory cell types, including B cells, CD8 T cells, and natural killer cells, was linked with susceptibility to severe bronchiolitis. Interestingly, previous studies suggest potential roles for *GSDMB* and *ORMDL3* expression in asthma pathobiology. Higher expression levels of *GSDMB* associated with asthma and increased *GSDMB* expression further affected genes involved in interferon signaling, major histocompatibility complex class I antigen presentation, and immune pathways [48, 49]. Moreover, the expression of *ORMDL3* was affected by rhinovirus infection [50]. Interestingly, the index variant of the current GWAS (rs9303281) was suggested to modulate *ORMDL3* expression and asthma risk in rhinovirus-treated airway epithelial cells [50]. Like *GSDMB*, increased expression of *ORMDL3* has been linked to interferon signaling and immune response in the context of asthma, and we suggest that similar regulatory pathways may play a role in susceptibility to severe bronchiolitis.

Our results indicate that the *CDHR3* variant rs6967330-A increases susceptibility to acute viral bronchiolitis in general and particularly if the causative agent is other than RSV. Multiple studies have linked the *CDHR3* variant rs6967330 with the risk of asthma [40, 42, 44, 45], and the variant has been associated with CDHR3 protein expression in the airway epithelial cells [42]. *CDHR3* is a receptor for rhinovirus C, and increased expression of CDHR3 modulated by rs6967330-A was associated with increased viral replication and susceptibility to rhinovirus C infection [42]. The current study found that the variant is associated with overall bronchiolitis. Thus, studying specific causative viruses of bronchiolitis and their interaction with *CDHR3* in molecular biological studies could provide further insight into bronchiolitis pathobiology.

The genetic correlation analysis found a high degree of shared genetic factors among severe bronchiolitis and asthma. Furthermore, asthma and related phenotypes were among the top traits associated with bronchiolitis in the pheWAS. This can be interpreted in 2 ways. First, common genetic determinants could indicate a shared genetic predisposition to bronchiolitis and asthma. Alternatively, severe bronchiolitis in early childhood may activate pathways and processes that increase the risk of subsequent asthma. Based on the knowledge of complex traits influenced by multiple environmental and genetic factors and their interaction, it could be that bronchiolitis serves as a marker of predisposition to asthma and participates in molecular pathways that promote the development of asthma.

This study has certain limitations. The analysis was performed with data from Finnish individuals; thus, the findings may not be generalizable to populations with different ancestry. The number of individuals with non-RSV and RSV bronchiolitis was limited because viral etiologies were recorded only during the *ICD-10* diagnoses. Thus, the subanalyses should be repeated in larger virus-specific populations. Furthermore, some controls without a history of LRI may not have been at risk for RSV infection during the most vulnerable period (ie, during the first 6 months of life). Moreover, there are no standardized criteria for the need of outpatient visit or hospitalization due to bronchiolitis, so the terms “bronchiolitis” and “severe bronchiolitis requiring hospitalization” are somewhat arbitrary. Finally, although this is the largest GWAS of bronchiolitis to date, the number of bronchiolitis-associated loci is still limited, hindering our ability to determine causal effects between severe bronchiolitis and asthma by mediation analysis.

In summary, the current study provides evidence of genetic associations and perhaps genetically mediated functional mechanisms for the *CDHR3* and *GSDMB* loci in severe bronchiolitis. The detected effects were larger in non-RSV vs RSV bronchiolitis. More fine-grained genetic and molecular biological studies in virus-specific bronchiolitis populations with sufficient sample sizes could be beneficial in specifying the effects of these loci. Based on the importance of non-RSV bronchiolitis for the risk of subsequent asthma, a vaccine protecting against rhinovirus infections in childhood could be of significance, as rhinoviruses are among the major causative agents of non-RSV bronchiolitis. Effective prevention of infant bronchiolitis could have effects on lifelong respiratory health by reducing susceptibility to asthma, provided that non-RSV bronchiolitis has effects on causal pathways of asthma. The results from our study demonstrate that there are shared genetic factors underlying the known association between severe bronchiolitis and later asthma.

## Supplementary Material

jiae467_Supplementary_Data

## Supplementary Data

Supplementary materials are available at *The Journal of Infectious Diseases* online (http://jid.oxfordjournals.org/). Supplementary materials consist of data provided by the author that are published to benefit the reader. The posted materials are not copyedited. The contents of all supplementary data are the sole responsibility of the authors. Questions or messages regarding errors should be addressed to the author.

## Notes

***Acknowledgments*.** CSC–IT Center for Science, Finland, is acknowledged for computational resources. We want to acknowledge the participants and investigators of the FinnGen study. The following biobanks are acknowledged for delivering biobank samples to FinnGen: Auria Biobank, THL Biobank, Helsinki Biobank, Biobank Borealis of Northern Finland, Finnish Clinical Biobank Tampere, Biobank of Eastern Finland, Central Finland Biobank, Finnish Red Cross Blood Service Biobank, Terveystalo Biobank, and Arctic Biobank. All Finnish Biobanks are members of BBMRI.fi infrastructure. Finnish Biobank Cooperative–FINBB is the coordinator of BBMRI-ERIC operations in Finland. The Finnish biobank data can be accessed through the Fingenious services managed by FINBB.

***Author contributions.*** Conceptualization: A. P., M. H., and M. R. Supervision: M. R. and M. H. A. P. carried out the formal analysis, investigation, visualization, and writing of the initial manuscript. FinnGen provided the study data, computational environment, and analysis tools for phenotyping and genome-wide association analyses. All authors critically reviewed the results and contributed to the final manuscript.

***Data availability.*** The GWAS summary data will be made available in the NHGRI-EBI Catalog of human GWASs upon publication with the accession numbers GCST90451658 (bronchiolitis), GCST90451659 (non-RSV bronchiolitis), and GCST90451660 (RSV bronchiolitis).

***Financial support*.** This work was supported by the Stiftelsen Alma och K.A. Snellman Säätiö (to A. P.); Sigrid Jusélius Foundation (to M. H.); and Foundation for Pediatric Research (to M. R.). The FinnGen project is funded by 2 grants from Business Finland (HUS 4685/31/2016 and UH 4386/31/2016) and the following industry partners: AbbVie Inc, AstraZeneca UK Limited, Biogen MA Inc, Bristol-Myers Squibb (and Celgene Celgene & Celgene International II Sàrl), Genentech Inc, Merck Sharp and Dohme LLC, Pfizer Inc, GlaxoSmithKline Intellectual Property Development Limited, Sanofi US Services Inc, Maze Therapeutics Inc, Janssen Biotech Inc, Novartis AG, and Boehringer Ingelheim International GmbH.
